# Citizenship Pressure as a Predictor of Daily Enactment of Autonomous and Controlled Organizational Citizenship Behavior: Differential Spillover Effects on the Home Domain

**DOI:** 10.3389/fpsyg.2019.00395

**Published:** 2019-02-28

**Authors:** Lynn Germeys, Yannick Griep, Sara De Gieter

**Affiliations:** ^1^Department of Work and Organisation Studies, KU Leuven, Leuven, Belgium; ^2^Research Group of Work and Organizational Psychology, Vrije Universiteit Brussel, Brussels, Belgium; ^3^Department of Psychology, University of Calgary, Calgary, AB, Canada; ^4^Division of Epidemiology, Stockholm University, Stockholm, Sweden

**Keywords:** organizational citizenship behavior, extra-role performance, citizenship pressure, work-home conflict, work-home enrichment

## Abstract

This study questions the exclusive discretionary nature of organizational citizenship behavior (OCB) by differentiating between autonomous OCB (performed spontaneously) and controlled OCB (performed in response to a request from others). We examined whether citizenship pressure evokes the performance of autonomous and controlled OCB, and whether both OCB types have different effects on employees’ experience of work-home conflict and work-home enrichment at the within- and between-person level of analysis. A total of 87 employees completed two questionnaires per day during ten consecutive workdays (715 observations). The results of the multilevel path analyses revealed a positive relationship between citizenship pressure and controlled OCB. At the within-person level, engaging in autonomous OCB resulted in an increase of experienced work-home conflict and work-home enrichment. At the between-person level, enactment of autonomous OCB predicted an increase in experienced work-home enrichment, whereas engaging in controlled OCB resulted in increased work-home conflict. The divergent spillover effects of autonomous and controlled OCB on the home domain provide empirical support for the autonomous versus controlled OCB differentiation. The time-dependent results open up areas for future research.

## Introduction

Organizational citizenship behaviors (OCB) are discretionary extra-role behaviors that go beyond an employee’s contractual job and role prescription (e.g., performing additional work tasks to help a colleague; Organ, 1988). Traditionally, research (see meta-analysis of Podsakoff et al., 2009) focused on the positive consequences of OCB for the organization (e.g., higher productivity, higher customer satisfaction) and employee (e.g., less absenteeism, less turnover intentions). However, scholars started to question the exclusive voluntary nature of OCB (e.g., for some theoretical papers see Vigoda-Gadot, 2006; Spector and Fox, 2010; Spitzmuller and Van Dyne, 2013; Grant and Bolino, 2016) and associated beneficial outcomes (e.g., Halbesleben et al., 2009, 2010; Turnipseed and Wilson, 2009; Gailliot, 2010; Weinstein and Ryan, 2010; Koopman et al., 2016; Spanouli and Hofmans, 2016; Liu et al., 2017; Yam et al., 2017; Gabriel et al., 2018). Self-determination theory (SDT; Deci and Ryan, 1985; Ryan and Deci, 2000) states that employees can experience different underlying reasons to engage in a similar behavior. Derived from SDT (Deci and Ryan, 1985), we draw on the differentiation between autonomous and controlled helping (Weinstein and Ryan, 2010), to argue that an employee might engage in OCB for (1) spontaneous, discretionary and volitionally reasons (i.e., autonomous OCB), or (2) to comply or satisfy an implicit or explicit request from colleagues or a supervisor (i.e., controlled OCB; Bateman and Organ, 1983; Hoffman et al., 2007). The perceived locus of causality to engage in a behavior (i.e., perceived reason for action; Deci and Ryan, 1985; Gagné and Deci, 2005) is conditional upon the extent to which one feels that one initiated the behavior oneself (i.e., internal) or acted in response to external factors beyond one’s control (i.e., external). In line with this differentiation, helping a colleague who’s struggling with heavy workloads for the inherently satisfying feeling would be categorized as autonomous OCB, whereas enacting the same behavior in order to satisfy the colleague’s request for help would be conceptualized as controlled OCB.

In many cases, the ideal employee is considered someone who not only meets all the requirements of the job description but is also someone who goes the extra mile (Williams, 2000). As a result, an employee can experience citizenship pressure, that is an individual’s subjective feeling of organizational or group pressure to engage in behaviors that are supposedly non-mandatory in nature, such as OCB (Bolino et al., 2010). In line with the focus of SDT (Deci and Ryan, 1985) to examine undermining work environments with the aim to redesign and optimize the working conditions, we examined the role of citizenship pressure, as a stable contextual variable, on the enactment of autonomous and controlled OCBs (Deci et al., 1994; Crant, 2000).

The majority of employees need to concurrently balance the demands and resources in the work and home domain. Scholarly attention focused on employees’ work-home interface and provided ample evidence of the importance of effectively managing the work and home domain for employees’ well-being and performance at work as well as at home (e.g., absenteeism, burnout, family and job satisfaction; Amstad et al., 2011). The work-home interface is characterized by an intra-individual transmission or spillover of demands or resources, behaviors, thoughts or emotions from work to home^1^ (Greenhaus and Powell, 2006). This spillover can be negative due to the incompatibility of the work demands (e.g., work overload, time pressure) with the employee’s home demands (e.g., house hold chores, child care), referred to as work-home conflict. On the contrary, the spillover can be positive when participating in the work role will improve an employee’s functioning in his/her home role (e.g., skills learned at work that are beneficial at home), referred to as work-home enrichment. In this respect, some scholars have found a positive effect for the enactment of OCB on employees’ home life (i.e., work-home enrichment; Kwan and Mao, 2011), whereas others found a negative effect (i.e., work-home conflict; Bolino and Turnley, 2005). These mixed findings raise the prevailing research question whether enactment of OCB is beneficial or detrimental for the employee’s home domain? In an attempt to further elucidate these mixed results, we examine the proposition of the SDT that the consequences of engaging in a specific behavior are based on the employee’s motivation to enact in this behavior (Ryan and Deci, 2000). In line with prior research findings, we support the idea that the consequences of OCB may depend upon the employee’s motive to employ OCB (Weinstein and Ryan, 2010; Dávila and Finkelstein, 2013; Spanouli and Hofmans, 2016).

This paper integrates the literatures on OCB, citizenship pressure and the work-home domain to extend the extant literature and to advance the theoretical understanding. First, we contribute to the OCB literature by empirically differentiating between autonomous and controlled OCB in line with SDT (Deci and Ryan, 1985). Furthermore, we will take the contextual work situation into account by examining whether employees’ subjective experience of overall citizenship pressure in the organization triggers or blocks the enactment of autonomous and controlled OCB. Second, we contribute to the literature on the work-home interface by simultaneously elaborating on the positive and negative spillover effects from OCB at work to the home domain. Regardless of the research finding that work-home conflict and enrichment can co-occur (Frone, 2003; Carlson et al., 2006), most previous studies investigated only the positive or the negative spillover effect. Third, and most importantly, we want to answer the prior inconsistent research findings linking OCB and employees’ (positive and negative) work-home interface. Thereto, we integrate this literature stream with the stream on the non-exclusive voluntary nature of OCB. We will examine whether engaging in OCB for substantially different motives, results in different spillover effects to the home domain. Addressing the aforementioned research question requires a shift away from traditional methods used in the study of OCB and the work-home interface. Our current understanding about the relationship between OCB and the work-home interface comes from studies that are cross-sectional in nature and which are characterized by mixed findings. However, recent empirical developments have questioned the validity of treating OCB and the work-home interface as static concepts. These studies suggested that both OCB and the work-home interface are dynamic constructs, changing on a day-to-day basis (Carlson et al., 2009; Bolino et al., 2012). Subsequently, we thus conducted a daily survey study which allows us to capture the meaningful within-person variability and day-to-day variation in OCB and the work-home interface. In line with prior studies that found different within- and between-person consequences of OCB (i.e., with counterproductive work behaviors; Dalal et al., 2009), we will examine whether the consequences of OCB on the home domain differ depending on (1) the motive to engage in OCB (i.e., autonomous and controlled) and (2) the level of analysis.

## Hypothesis Development

### Citizenship Pressure

Prior research conceptualized citizenship pressure as an employee’s general feeling of being pressured to go above and beyond formal work tasks (Bolino et al., 2010). Citizenship pressure is similar to an OCB pressuring organizational climate, although it is focused on an employee’s general experience instead of the shared experience among employees. Nevertheless, just as organizational climate only changes over years (Schneider et al., 2013), citizenship pressure is conceptualized as stable within employees and therefore does not warrant a repeated measurement approach (Bolino et al., 2010). Experiencing citizenship pressure can operate as a stable external controlling force that implicitly or/and explicitly indicates the desirability of different behaviors within the organization or team (Meyer et al., 2010). This perspective on citizenship pressure acting as a situational strength was supported by Morrison (1994) who noted that supervisors encourage and prescribe their employees to engage in OCBs. Due to supervisors’ tendency to define task performance in a broader sense than employees do, they categorize several OCBs as being inherently part of employees’ contractual responsibilities. As a result, supervisors might create a work environment in which an employee feels that it is expected to take up extra tasks and responsibilities at work to be seen as a good employee (Bateman and Organ, 1983; Vigoda-Gadot, 2006).

It is important to note that the probability to engage in a particular behavior is determined by the interaction between situational influences (e.g., situational strength) and individual differences (e.g., motivation; Cronbach, 1957). Among others, Deci and Ryan (1985, 2000) suggested that pressure induces a work context that undermines—though not eliminates—an employee’s free choice to perform intrinsically regulated behavior. Congruent with this line of reasoning, Niemiec and Ryan (2009) found that teachers who experienced pressure to comply with their job description were less likely to engage in autonomously motivated behavior (i.e., autonomous OCB), which can be explained by a shift from an internal locus of causality to an external locus of causality. Likewise, Reeve and Deci (1996) found that participants’ intrinsic motivation decreased in the experimental condition characterized by a competitive and pressuring interpersonal context. In a similar vein, we argue that experiencing citizenship pressure at work, will diminish an employee’s autonomously motivated enactment of OCB.

Hypothesis 1: Citizenship pressure is negatively related to the enactment of autonomous OCB.

Based on the suggested characteristic of citizenship pressure to undermine intrinsically regulated behavior and to shift the locus of causality externally, we posit that experiencing citizenship pressure will elicit controlled forms of behavior (Deci and Ryan, 2000). Students were more likely to become controlled motivated when encountering a controlling and pressuring, compared to an autonomy-supportive, teaching style (De Meyer et al., 2014). In other words, the controlling and pressuring school environment acted as a situational strength that induced a shift from an internal to an external perceived locus of causality. As a consequence, it evoked controlled motivation among students. Moreover, Bolino et al. (2010) conceptualized citizenship pressure as a job demand that represents the pressures an employee experiences to engage in OCB. In response to experienced job pressures, employees feel incentivized by outside forces to devote more time and energy at work (LePine et al., 2002). Likewise, we hypothesize that employees who experience citizenship pressure will increase their enactment of controlled OCB.

Hypothesis 2: Citizenship pressure is positively related to the enactment of controlled OCB.

### Autonomous OCB

Typically, scholars found support for the beneficial effects of autonomous work behaviors on the environment (e.g., successful work environment; Frese et al., 2007) as well as on the performer (e.g., career success; Seibert et al., 2001; Grant and Ashford, 2008). According to SDT (Deci and Ryan, 1985), spontaneously going beyond the formal job requirements without feeling obligated (i.e., autonomous OCB) will result in the acquisition of resources (Grant et al., 2011). This concept is supported by the expansionist hypothesis stating that resource generation at work (e.g., skills) will positively impact the functioning of the employee in his/her home role (Marks, 1977; Barnett and Hyde, 2001; Greenhaus and Powell, 2006). Ryan and Deci (2000) found that autonomously motivated behavior compared to controlled behavior, resulted in increased levels of self-esteem, confidence and well-being. Likewise, we assume that the inherently satisfying and resources-gaining characteristics (e.g., self-worth, sense of personal control) of autonomous OCB will positively spill over from work to home roles (i.e., work-home enrichment; Greenhaus and Powell, 2006). Therefore, we hypothesize that engaging in autonomous OCB at work will increase the experience of work-home enrichment at home.

Hypothesis 3: Employees’ enactment of autonomous OCB at work is positively related to their experience of work-home enrichment at home.

### Controlled OCB

Controlled work behaviors are behaviors that are elicited by someone (e.g., supervisor) or something (e.g., a situation; Frese and Fay, 2001). In line with SDT (Deci and Ryan, 1985), previous studies found a decline in beneficial outcomes at work (e.g., number of job offers; Grant et al., 2011) and available resources (e.g., time, energy; Muraven, 2008) when an employee’s behavior shifted from being internally to externally regulated behavior. In addition, the scarcity hypothesis states that enactment of controlled OCBs at work consumes resources. Hence, these resources are no longer available to be used in the home domain (Edwards and Rothbard, 2000). More precisely, the incompatibility of the work (e.g., approaching deadline) and home (e.g., house hold chores) demands escalates inter-role conflict when an employee invests the limited resources (e.g., time and energy) in the work role and consequently can no longer apply these resources to the home role (Edwards and Rothbard, 2000). Because employees perform controlled OCBs in response to an external perceived locus of causality, we posit that the resource-depleting characteristics of controlled OCB will negatively spill over from work to home roles (i.e., work-home conflict; Greenhaus and Beutell, 1985). Consequently, we hypothesize that engaging in controlled OCB at work will increase the experience of work-home conflict at home.

Hypothesis 4: Employees’ enactment of controlled OCB at work is positively related to their experience of work-home conflict at home.

Drawing on the abovementioned rational (i.e., hypothesis 1 and 3), autonomous OCB might operate as a mediator of the relationship between citizenship pressure experienced at work and work-home enrichment experienced at home. Specifically, citizenship pressure experienced at work can undermine an employee to autonomously engage in OCB (i.e., hypothesis 1). In turn, less enactment of autonomous OCB will prevent acquiring resources that could benefit the home domain, or in other words would hinder an employee to experience work-home enrichment (i.e., hypothesis 5). Taken together, one possible way through which the negative spillover from citizenship pressure encountered at work to work-home enrichment takes place is by a lack of enactment of autonomous OCB. Consequently, we hypothesize that:

Hypothesis 5: Employees’ autonomous OCB mediates the negative relationship between citizenship pressure experienced at work and work-home enrichment experienced at home.

In a similar vein, building on the abovementioned rational (i.e., hypothesis 2 and 4), the relationship between citizenship pressure experienced at work and work-home conflict experienced at home might be mediated by controlled OCB. Specifically, citizenship pressure experienced at work can instigate an employee to engage in OCB for controlled motives (i.e., hypothesis 2). In turn, enactment of controlled OCB might consume resources which can no longer be invested in the home domain, or in other words would increase an employee’s experience of work-home conflict (i.e., hypothesis 4). Taken together, one possible way through which the positive spillover from citizenship pressure encountered at work to work-home conflict experienced at home might take place is via the enactment of controlled OCB. Consequently, we hypothesize that:

Hypothesis 6: Employees’ controlled OCB mediates the positive relationship between citizenship pressure experienced at work and work-home conflict experienced at home.

## Materials and Methods

### Procedure

We contacted individual Belgian employees from different sectors via a snowball sample. During a personal conversation with each respondent, we explained the purpose of the study, stressing the discretionary nature of participation, the possibility to withdraw from the study and the confidential treatment of the data. Additionally, we provided each respondent with written information about the study, meaning that oral and written informed consent was offered. We interpreted indication of willingness to participate as informed consent. No incentives were provided for participation in the research. The Ethics Committee of the authors’ university exempts studies from ethical approval when they are non-invasive and harmless.

We asked our respondents to complete a single general survey, prior to completing two daily surveys per day for 10 consecutive workdays. All surveys were in Dutch, all survey items were translated to Dutch and colleagues back-translated the items to English. Inconsistencies between the translation and back-translation were discussed and resolved. We instructed our respondents to fill out the first daily survey at the end of each workday and asked them to fill out the second daily survey right before bedtime. For their convenience, we instructed them to keep the first survey booklet at the office and to put the second survey booklet on their nightstand at home. It was emphasized that they were not required to fill out the surveys on days they did not work. Additionally, we only included employees who completed more than three out of the ten dyads of daily surveys in a timely manner (i.e., completed on the requested time and day according to their self-reported time stamps) to minimize the effects of recollection bias. This daily survey design was chosen since it reduces the retrospective bias of more traditional survey studies (Reis and Gable, 2000) and allows us to account for the situational and temporal context when studying feelings, cognitions, and behaviors (Reis and Gable, 2000). Overall there were 151 missing observations (out of 870) of the first booklet (compliance rate = 82.6%) and 155 of the second booklet (compliance rate = 82.2%). Data were fully anonymized prior to the analyses.

### Respondents

A total of 87 Belgian employees participated in our study. Nearly half of the respondents were male (52%) with an average age of 34.89 years (*SD* = 11.92, range: 22–61 years). All respondents obtained at least a secondary school degree. The majority worked full-time (85%) and were employed in their current function for about 5.70 years (*SD* = 6.74). Approximately half of the respondents were cohabiting (53%) and had at least one child (46%). Traditionally, that is in cross-sectional or longitudinal research, the number of respondents serves as the sole unit of analysis. However, as our study has two levels of analysis, we have two units of analysis. For the within-person level, the unit of analysis equals ‘dyads of daily survey entries’ rather than ‘respondents’ (Conway and Briner, 2002). As a result, the sample size contains 715 observations (87 respondents x a maximum of 10 dyads of daily survey entries), or an average of 8.25 completed dyads of daily surveys per respondent. For the between-person level, the unit of analysis was 87 respondents. In a multilevel design, Browne and Draper (2000) showed that with more than 48 respondents, maximum likelihood estimation produced good variance estimations. More recently, Maas and Hox (2005) found that level two sample sizes exceeding 30 are sufficiently large to produce non-biased estimates and accurate estimations of standard errors and fixed effects.

### Measures

#### General Survey Measures

We used the general survey to collect demographic information and citizenship pressure. *Citizenship pressure* was measured with the eight-item scale of Bolino et al. (2010). Respondents rated these items on a 5-point Likert scale ranging from “Never feel pressured to” (1) to “Always feel pressured to” (5). The scale includes items such as “Simply doing your formally prescribed job duties is not enough to be seen as a good employee in this organization.”

#### First Daily Survey (End of Workday)

*Autonomous and controlled OCB* were measured with a newly developed and pilot-tested measurement (developmental process information is available in the Supplementary Data Sheet 1). The scale instruction was formulated to capture the daily time frame (e.g., adding ‘today’ to the item; for a similar approach see Ilies et al., 2007). Respondents were provided with the following instruction: “Indicate whether you displayed each of the behaviors below today. Mark the column ‘Out of my own initiative’ if you engaged in the behavior spontaneously, without someone asking you. Mark the column ‘Elicited’ if you engaged in the behavior because someone expected, urged or explicitly asked you to. If you did not display the behavior today, mark ‘Behavior not enacted’.” A sample item of our final 20-item scale (a complete list of all items is available from the first author upon request) is: “Today I took over tasks of my colleague(s)/supervisor who had heavy workloads.” An employee’s scale score for autonomous and controlled OCB for a particular day was the sum of the column marks of “*Out of my own initiative*” and “*Elicited*,” respectively. Note that respondents could indicate that they performed the same behavior (i.e., item) on the same day once out of their own initiative and once elicited. In other words, they were not forced by the instruction, nor by the survey to choose between both.

#### Second Daily Survey (Before Bedtime)

*Work-home conflict and enrichment* were measured with items from established validated scales that could occur on a daily level (for a similar approach see Dalal et al., 2009). Hence, work-home conflict was measured with five items from Netemeyer et al. (1996). A sample item was: “Today, my job produced strain that made it difficult to fulfill home duties.” *Work-home enrichment* was measured with five items from the scale of Carlson et al. (2006). A sample item was: “Today, my work provided me with a sense of success and this helped me to be a better family member.” Respondents rated these items on a 5-point Likert scale ranging from “strongly disagree” (1) to “strongly agree” (5).

*Time-lagged variables* were created for work-home conflict and work-home enrichment. These time-lagged variables were created by taking the score of the same individual on the same variable during the previous day, and they were only created when a respondent filled out at least two consecutive daily surveys. These time-lagged variables were used to control for potential confounding effects due to autocorrelations (i.e., the cross-correlation of a variable with itself over the course of the 10 consecutive working days) and to check for stability versus change in the outcome variables (Bliese and Ployhart, 2002).

### Data Analysis

Given the nested structure in our data (i.e., dyads of daily surveys nested within individuals), we estimated the intraclass correlation coefficients (ICC) of autonomous and controlled OCB, and work-home conflict and enrichment to assess the need for a multilevel modeling approach (Hox, 2010). Results indicated that a substantial proportion of the variance in these variables (ICC values are 0.65, 0.65, 0.54, and 0.60, respectively) could be attributed to within-person differences, supporting a multilevel approach (Marcoulides and Schumacker, 2009). We opted for a multilevel path analysis because it is especially suited for complex models in which change in a given variable is not influenced by significant baseline differences. Furthermore, path analysis allows for all outcomes to be correlated at each point in time and to simultaneously estimate multiple path coefficients (Lleras, 2005). Hence, we estimated a multilevel path analysis using Mplus version 7.1 (Muthén and Muthén, 2012), in which we estimated a two-level path model at the within-person level, and the between-person variability in order to retain the distinct within-person variability (Maas and Hox, 2005).

We separated the within- and between-person effects by relying on the unconflated 2-1-1 mediation model thereby avoiding the single conflation slope bias (Zhang et al., 2009; Preacher et al., 2010). Prior to specifying the between-person (i.e., level two) × within-person (i.e., level one) part of the two-level path model, the level two predictor variable (i.e., citizenship pressure) was grand-mean centered. This rescaling of the level two predictor variable facilitates the interpretation of the results (Enders and Tofighi, 2007; Preacher et al., 2010). Specifically, we specified the between-person part of the two-level path model reflecting the direct relationship of citizenship pressure on autonomous and controlled OCB (i.e., hypotheses 1 and 2). Next, the level-1 variables (i.e., autonomous/controlled OCB and work-home conflict/enrichment) were decomposed into within- and between-person level relationships as recommended by Zhang et al. (2009) and McNeish (2017). Prior to specifying the within-person part of the two-level path model we person-mean centered the level-1 predictor variables. In this part of the model we predicted the daily associations between autonomous and controlled OCB, and work-home conflict and enrichment. For between-person level analyses we included an individual’s mean (i.e., autonomous and controlled OCB). In this part of the model we predicted the general associations between autonomous and controlled OCB, and work-home conflict and enrichment (i.e., hypotheses 3 and 4). Moreover, we examined the between-person mediation effects (i.e., hypothesis 5 and 6). We compared the balance between the number of parameters (i.e., model complexity) and the fit of the model to the data (i.e., Bayesian Information Criterion or BIC) of a full and a partial mediation model. According to the BIC values, the full mediation model yielded a superior fit to the data (BIC_fullmediation_ = 7892.84 < BIC_partialmediation_ = 7898.14; Aiken and West, 1991). This result is in line with the non-significant Chi-square difference test between the full and partial mediation model [*X*^2^_diff_(2) = 5.92, *p* > 0.05] indicating that the more parsimonious (i.e., full mediation) model is preferred. Consequently, we rely on the full mediation model when discussing the results. To control for serial dependency and to predict change in the mediator and outcome variables of our repeated measures data we included the time-lagged variables (i.e., autocorrelations for each variable; Bliese and Ployhart, 2002). For days without prior day’s values no prediction was made for that specific day and the data on that day were treated as missing, but these days were used as predictors for the next day (for a similar approach see Ohly et al., 2010). Maximum likelihood was used with robust standard errors as estimator in the path analysis. Our parameters were standardized estimates to facilitate the interpretation of our results (Hox, 2010).

## Results

### Internal Consistency Reliability

The internal scale reliability was assessed by estimating the level-specific omega coefficients since single-level estimates of reliability, such as Cronbach alpha coefficients, do not accurately reflect a scale’s actual reliability when variance exists at multiple levels (i.e., within- and between-person variance; Geldhof et al., 2013). The internal between-person reliability of the scale for citizenship pressure (ω = 0.91, 95%CI [0.88, 0.94]), work-home conflict (ω = 0.94, 95%CI [0.91, 0.97]), and work-home enrichment (ω = 0.89, 95%CI [0.85, 0.94]) were satisfactory. In addition, the internal within-person reliabilities of the scales for work-home conflict (ω = 0.78, 95%CI [0.72, 0.84]), and work-home enrichment (ω = 0.64, 95%CI [0.56, 0.71]) were acceptable. Note that internal consistency reliability coefficients for autonomous and controlled OCB were not reported since these are formative constructs. Items in such formative constructs do not necessarily share high inter-item correlations because one does not necessarily have to engage in each of the specific behaviors simultaneously for the latent construct to emerge (which is the case with a reflexive construct). However, as the calculation of internal consistency reliability coefficients requires such high inter-item correlations, the estimation of internal consistency reliability coefficients is inappropriate for formative constructs (Diamantopoulos and Siguaw, 2006; Coltman et al., 2008).

### Confirmatory Factor Analysis

A multilevel CFA was performed, in which we specified work-home conflict and enrichment at the within-person and between-person level, whereas citizenship pressure was only specified at the between-person level. Overall, our model achieved a good to reasonable fit (RMSEA = 0.03, CFI = 0.90, TLI = 0.88, SRMR_within_ = 0.05, SRMR_between_ = 0.15). Additionally, each item loaded significantly and in the expected direction onto its respective latent factor. Note that a CFA for autonomous and controlled OCB at the within-person level was not conducted nor appropriate since, on a daily level, these are formative constructs (Coltman et al., 2008). Formative constructs assume that the latent construct is formed bottom-up (the non-interchangeable items drive the emergence of the latent construct) instead of being present (the construct is present and can be measured with interchangeable items; Borsboom et al., 2003, 2004). Although conducting a CFA is inappropriate at the within-person level, we did compare the BIC value of the hypothesized two-factor OCB model at the between-person level (BIC = 46935.74) with the single-factor OCB model (BIC = 49447.35). This comparison revealed that the hypothesized two-factor OCB model fit the data better than the single-factor OCB model (Aiken and West, 1991). Furthermore, all items in the two-factor model loaded significantly and in the expected direction on their corresponding latent factor. Finally, to demonstrate the empirical distinction between citizenship pressure, autonomous OCB, and controlled OCB, we conducted a CFA at the between-person level and demonstrated that a three-factor model (RMSEA = 0.05, CFI = 0.91, TLI = 0.90, SRMR = 0.07), in which items load onto their corresponding latent factor, fit the data best.

### Descriptive Statistics

Table 1 reports the means, between- and within-person standard deviations, between-person correlations and within-person correlations of the variables under study. The means, between-person standard deviations and correlations were computed on the aggregated dataset and the within-person standard deviations and correlations on the person-centered dataset.

**Table 1:** Means, standard deviations, between- and within-person correlations among the focal variables.

	*M*	*SD*_between_	*SD*_within_	1	2	3	4	5
(1) Autonomous OCB	8.02	3.99	2.67	–	−0.24^∗∗∗^	0.12^∗∗^	0.12^∗∗^	–
(2) Controlled OCB	1.63	2.12	1.41	−0.10^∗∗^	–	0.07	0.03	–
(3) Work-home conflict	2.06	0.72	0.58	0.08^∗^	0.24^∗∗∗^	–	0.02	–
(4) Work-home enrichment	2.28	0.62	0.44	0.15^∗∗∗^	0.11^∗∗^	0.20^∗∗∗^	–	–
(5) Citizenship pressure	2.48	0.86	–	−0.01	0.16^∗∗∗^	0.20^∗∗∗^	0.14^∗∗∗^	–

*^∗^p < 0.05, ^∗∗^p < 0.01, ^∗∗∗^p < 0.001. Between-person correlations are presented below the diagonal (N = 87). Within-person correlations are presented above the diagonal (N = 715).*

### Hypothesis Testing

**FIGURE 1:**
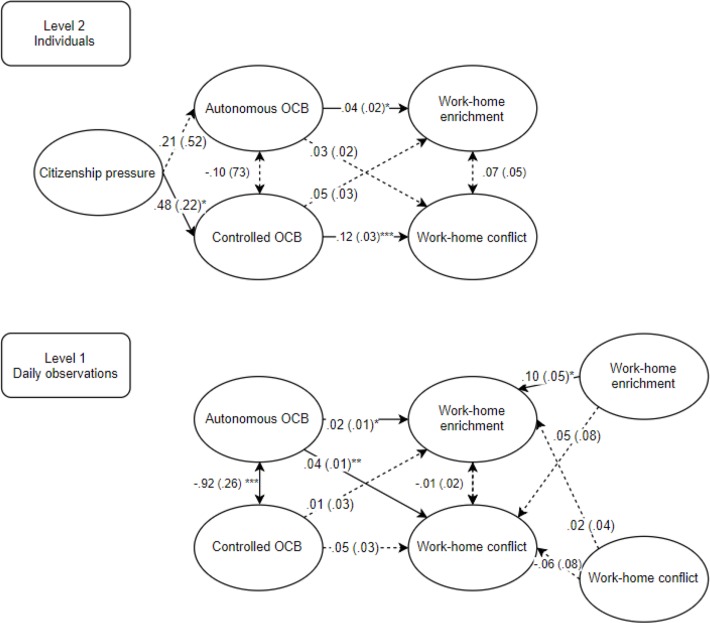
Results of the two-level path analysis for relations between citizenship pressure, autonomous and controlled OCB, and work-home conflict and enrichment on the between-person **(top)** and within-person **(bottom)** level. *N* = 87; *Within-person level N* = 715; β coefficients are standardized; Standard errors are between parentheses; ^∗^*p* < 0.05, ^∗∗^*p* < 0.01, ^∗∗∗^*p* < 0.001.

Figure 1 represents the standardized results of the two-level path analysis used to test our hypotheses. We found no significant relationship between general feelings of citizenship pressure and the enactment of autonomous OCB (β = 0.21, *ns.*). Hence, these results did not support Hypothesis 1. Next, in support to Hypothesis 2, general feelings of citizenship pressure had a positive effect on the daily enactment of controlled OCB (β = 0.48, *p* < 0.05). Because established between-person relationships do not always transfer to the within-person level (Zhang et al., 2009), we examined the relationship between autonomous and controlled OCB, and work-home conflict and enrichment on both levels of analysis. At the within-person level, support was found for Hypothesis 3, namely for a positive relationship between enactment of autonomous OCB and experiencing work-home enrichment (β = 0.02, *p* < 0.05) on a daily basis. Although not hypothesized, we also found that enactment of autonomous OCB was positively related to work-home conflict (β = 0.04, *p* < 0.01) on a daily basis. However, support for a direct effect of enactment of controlled OCB on the experience of work-home conflict (β = 0.05, *ns.*) on a daily level was not found; thereby not supporting Hypothesis 4. At the between-person level, support was found for Hypotheses 3 and 4. Specifically, enactment of autonomous OCB was positively related to experiencing work-home enrichment (β = 0.04, *p* < 0.05), whereas enactment of controlled OCB was positively related to experiencing work-home conflict (β = 0.12, *p* < 0.001). We found no support for the mediating role of autonomous OCB on the relation between citizenship pressure and work-home enrichment (β = 0.01, *ns.*; Hypothesis 5). In addition, controlled OCB did not significantly mediate the relation between citizenship pressure and work-home conflict (β = 0.06, *ns.*; Hypothesis 6).

## Discussion

Although OCB was originally defined as a discretionary behavior (Organ, 1988), some scholars questioned the voluntary nature of OCB (e.g., compulsory citizenship behavior; Vigoda-Gadot, 2006; Turnipseed and Wilson, 2009; Spector and Fox, 2010). In the current study, both perspectives on OCB were integrated. To that end, we theoretically differentiated OCB in autonomous OCB and controlled OCB. Hence, we differentiated between an internal (i.e., enacted discretionary) and external (i.e., enacted in response to someone’s implicit or explicit request) perceived locus of causality to initiate OCB. In addition, we investigated the predictive role of the work environment—measured as citizenship pressure—on the enactment of autonomous and controlled OCB. Furthermore, we investigated and found that different forms of OCB (i.e., autonomous and controlled OCB) related to both work-home conflict and enrichment at the within- and between-person level. We did not find support for the mediation role of autonomous nor of controlled OCB on the relation between citizenship pressure and the work-home interface. Overall, we contribute to the OCB literature by empirically demonstrating the discriminative power of the differentiation between autonomous and controlled OCB in predicting both the positive and negative spillover from the work to home domain. This is especially relevant because the work-home interface in itself is an important predictor of health outcomes and work behavior (e.g., Amstad et al., 2011). Previous research found that work-home conflict relates mainly to negative outcomes, such as lower work engagement (Fiksenbaum, 2015), whereas work-home enrichment correlates mainly with positive outcomes, such as well-being (Nordenmark, 2004). Moreover, our findings provide support for different inter- and intra-individual effects of enactment of autonomous and controlled OCB on the experience of work-home conflict and enrichment, suggesting different time-dependent underlying mechanisms. However, we acknowledge that the standardized coefficients are quite small in magnitude, despite being significant. As such, our results should be interpreted with caution.

### Theoretical Implications

We found partial support for the SDT (Deci and Ryan, 1985) rationale that an external controlling work environment—in our study characterized by high citizenship pressure—would undermine the enactment of intrinsically regulated behavior (i.e., autonomous OCB), whereas it would stimulate the enactment of extrinsically regulated behavior (i.e., controlled OCB; Deci and Ryan, 1985, 2000). Our findings indeed indicated that employees who perceived citizenship pressure, were more likely to enact in controlled OCB. However, a negative relationship was not found between the experience of citizenship pressure and the enactment of autonomous OCB. In a similar vein, some researchers (e.g., Marinak and Gambrell, 2008) found inconsistent results between environmental pressures and the enactment of autonomous behavior. This might suggest that engaging in autonomous behaviors can happen independently of external controlling forces, such as citizenship pressure for example, by experiencing the fulfillment of basic psychological needs (i.e., need for autonomy, competence, and relatedness). Deci and Ryan (2002) found that employees who were satisfied with their basic psychological needs were more likely to engage in autonomously motivated behavior.

With regard to the consequences of OCB outside the work domain, our results indicated that, on a daily basis, an employee who spontaneously enacted OCB (i.e., autonomous OCB) may be more likely to have experienced a positive spillover effect from work to home (i.e., work-home enrichment). This finding aligns with previous studies stating that behaviors that are autonomous in nature will benefit the employee who is enacting in that behavior (Grant and Ashford, 2008), as well as with SDT (Deci and Ryan, 1985) as intrinsically initiated behaviors at work presumably yield resources which in turn could be applied in the home role. However, autonomous OCB also related to work-home conflict on a daily basis. Although not hypothesized, these findings can be explained in the light of the resources investment principle of the Conservation of Resources Theory (Hobfoll, 2001) stating that employees invest resources to gain new resources (e.g., Ng and Feldman, 2012). Bergeron (2007) found that due to individuals’ limited pool of resources, resource allocation to engage in OCBs prevents these resources to be invested in other domains (family activities and leisure activities). Prior research found support for the energy consuming aspect of engaging in OCB, regardless of the underlying motive (Lanaj et al., 2016). During a specific work day, expending resources at work depletes an individual’s energy, leaving fewer resources to expending on other life experiences, with potential professional and personal costs (Bolino et al., 2013). Specifically, the invested resources needed to engage in autonomous OCB at work are no longer available to invest in the home domain, which can potentially result in work-home conflict. Furthermore, this finding confirms previous arguments of, among others, Boz et al. (2009) stating that an employee can experience work-home conflict and enrichment concurrently (e.g., during the same day) since they are distinct, non-mutually exclusive constructs that are not situated on the opposite end of the same continuum (Frone, 2003). In other words, these findings support the coexistence of the expansionist and the scarcity hypothesis, namely that the spillover from enactment of autonomous OCB at work to the home domain can simultaneously generate and consume resources during the same day. In a practical sense this implies that enacting OCB out of one’s own initiative can consume time or energy that can no longer be devoted to home activities during the same day, which in turn leads to work-home conflict. Conversely, enactment of autonomous OCB can allow one to acquire a good self-concept or new skills, which could improve work-home enrichment during the same day, as well as over longer periods of time. Hence, it seems that our findings suggest different time-dependent effects; whereas at the momentary level enactment of autonomous OCB seems positively related to both work-home conflict and enrichment, engaging in autonomous OCB may be largely beneficial for the home domain over longer periods of time. This alternative explanation is supported by our results at the between-person level as they point out that the enactment of autonomous OCB is positively related to work-home enrichment, whereas it is unrelated to work-home conflict. We believe that this between-person effect might be due to the additive positive daily effects of engaging in autonomous OCB for the home domain (i.e., learning skills, positive mood, and sense of self-worth), while the daily negative effects of autonomous OCB for the home domain might level out over time.

Subsequently, enactment of controlled OCB was not significantly related to the experience of work-home conflict on a daily basis, whereas, enactment of controlled OCB leads to a negative spillover from the work to the home domain (i.e., work-home conflict) over longer periods of time, as exemplified by our between-person results. One possible explanation for the non-significant relationship at the daily level can be found considering the low variance of the daily occurrence of controlled OCB. This low variance suggests that an employee makes very little changes in the enactment of controlled OCB over the course of ten workdays. Thus, we are far less likely to find significant evidence for the proposed positive relationship between controlled OCB and work-home conflict at the within-person level. On the between-person level, our finding aligns with the tenets of SDT (Deci and Ryan, 1985; Muraven, 2008; Grant et al., 2011) as extrinsically initiated behaviors at work increase the experience of work-home conflict, presumably by depleting resources which in turn can no longer be used in the home role. These different time-dependent effects can be explained by drawing from the underlying assumptions of the sleeper model used in stress research to explain how a stressor relates to the coming about of strain (Frese and Zapf, 1988). Specifically, they state that the consequences of a specific behavior do not always occur instantaneously but might add up over time before they translate into negative effects.

Furthermore, experiencing work-home conflict during 1 day, did not seem to influence the likelihood of experiencing work-home conflict or enrichment during the next day. However, experiencing work-home enrichment during 1 day did influence the levels of experienced work-home enrichment during the next day, but did not influence the level of experienced work-home conflict. The significant autoregressive effect of work-home enrichment would suggest that an increase in the experienced levels of work-home enrichment on a given day would predict increases in experienced enrichment during the following day. This finding is in line with the broaden and build theory (Fredrickson, 2004) which stipulates that over time the acquired skills and resources build up and will eventually translate into sustained positive emotions or experiences, such as work-home enrichment.

### Limitations

Notwithstanding the methodological and theoretical contributions, our research has limitations that deserve further attention. First, the self-reported nature of our measures might raise concerns about social desirability and common method variance (Podsakoff et al., 2003). However, we assume that these threats only scarcely influenced our results since we measured the predictor and outcome variables at different points in times (i.e., across two daily surveys; Podsakoff et al., 2003) and eliminated between-person variance by person-centering the predictor variables. By doing so, we thus also eliminated variance caused by individual response tendencies (Ilies et al., 2010). Concerning the self-reported nature of the OCB measures, meta-analytic findings support the convergence between self- and other-rated data (Carpenter et al., 2014). Furthermore, we question the possibility for supervisors and/or colleagues to capture the day-to-day variance in OCB and, most importantly, the motive of an employee to enact autonomous or controlled OCB. Notwithstanding this concern, we encourage further research to include other-ratings of OCB in the light of the organizational trend to rely on teamwork (Mathieu et al., 2017).

A second limitation concerns our sampling strategy. We recruited respondents by means of a snowball sample, which potentially resulted in a sample that is not representative of the general population. However, meta-analytic findings (Wheeler et al., 2012) suggest slightly lower effect sizes and correlations in snowball samples compared to non-snowball samples, whereas the same overall conclusions could be drawn from both samples. Hence, based on their meta-analytical findings, the use of a snowball sample, such as in this study, would have resulted in more conservative estimates of the relationships between the variables under study.

A third limitation concerns the use of self-reported time stamps in our paper-and-pencil surveys. Studies examining the work-home interface often rely on paper-and-pencil booklets to avoid attrition due to assessing variables at work as well as at home (for a similar approach see Volman et al., 2013). Although we chose this approach to allow respondents without a work laptop or internet access at home to participate in the study, we cannot verify the truthfulness of their indicated time stamps. However, we took some steps to minimize the potential that respondents could untruthfully indicate time stamps. That is, we instructed our respondents to leave the survey blank in case they forgot to fill it out. As some respondents did while they did indicate that they went to work that day, we are relatively confident that the self-reported time stamps are trustworthy. Moreover, participation was strictly voluntary with no incentive contingent on completion of the surveys. Hence, respondents have little external motivation to retrospectively complete the surveys. However, to objectify the time and day of survey completion, we recommend future research to rely on electronic surveys with automatic time stamps.

### Suggestions for Future Research

The current study opens up new avenues for further research. Our results point to the importance of differentiating between autonomous and controlled OCB when examining the influence of OCB on non-work related outcomes. Moreover, we also recommend including work related outcomes because the differentiation in OCB could also explain why some researchers found a negative (e.g., MacKenzie et al., 1999), and others found a positive (e.g., Griffin et al., 2000) relationship between OCB and task performance. In addition, it could be valuable to examine the spillover from the home to the work domain; an employee’s home life could possibly influence the enactment of autonomous and controlled OCB at work.

For reasons of parsimony, we did not differentiate between the different types of work-home conflict (i.e., time-, strain- and behavior-based conflict; Greenhaus and Beutell, 1985) and enrichment (i.e., capital-, affect- and developmental-based enrichment; Carlson et al., 2006). However, future research could examine whether autonomous and controlled OCB relate differently to the different types of work-home conflict and enrichment.

Finally, studies identifying situational moderators of the relationship between citizenship pressure and the enactment of controlled OCB are lacking. As our results indicated that experiencing citizenship pressure aggravates the enactment of controlled OCB which in turn might lead to experiencing work-home conflict in the long run, it might be useful to explore variables that could potentially buffer the negative effect of citizenship pressure. One such variable could be work-home friendly culture at work, as previous research found positive effects of such an organizational culture on an employee’s enactment of discretionary OCB (Allen, 2001; Bragger et al., 2005). In addition, although researchers conceptualized and measured citizenship pressure and work-home friendly culture at work as stable variables, it might be interesting to examine the ideal time lag to capture fluctuations in these variables.

### Practical Implications

Given the importance of OCB in today’s work environment, understanding the temporal relationship with citizenship pressure and the work-home interface provides policy makers with a powerful instrument. To begin with, our results underlined the negative effect of citizenship pressure, especially since citizenship pressure incentivized the enactment of controlled OCB, which in the long run is related to work-home conflict. Therefore, it seems valuable for organizations to assess the level of citizenship pressure that their employees experience and try to reduce these levels. If employees perceive that their organization is forcing them to engage in OCB, we would deem it beneficial to improve the work climate. This could, for example, be achieved by adhering to less hierarchically structured work environments (Frese and Fay, 2001; Grant and Ashford, 2008) or by fostering an autonomy supportive climate (Deci and Ryan, 2000). By doing so, the organization creates an environment in which persons with authority, such as managers or coordinators, can take the perspectives of employees into account, offer relevant information and opportunities to choose from, encourage initiative, provide optimal challenges and positive feedback, and facilitate a secure environment for social interactions. In line with the theoretical tenets of SDT (Deci and Ryan, 1985), such an environment is generally associated with more intrinsic motivation, greater interest, less pressure and tension, more creativity, and more flexibility, which most likely will foster the enactment of autonomous OCB, opposed to controlled OCB.

Secondly, it is important to raise employees’ awareness of the potential consequences of going the extra mile at work. Although enactment of autonomous behaviors can have negative consequences in the short run, our study also highlights immediate positive outcomes for the home domain as well as long-term positive outcomes. Therefore, we recommend that organizations promote the enactment of autonomous behaviors and to empower their employees by creating an organizational climate high on autonomy and mutual trust (Frese and Fay, 2001; Grant and Ashford, 2008). Furthermore, our study highlighted that regularly enacting in controlled OCB is associated with an increase in work-home conflict. When an employee feels instigated to engage in OCB it’s worthwhile to set boundaries and discuss them with the supervisor. In this respect, we highlight the need of fostering supervisors’ acknowledgment to lead by example; that is not expecting anything in return when helping a colleague and refraining from urging one’s subordinates to engage in OCB.

## Conclusion

Our study contributes to the ongoing debate on whether OCB is always performed voluntarily and has only beneficial consequences (e.g., Spector and Fox, 2010; Grant and Bolino, 2016; Liu et al., 2017; Yam et al., 2017; Gabriel et al., 2018). We differentiated OCB in an autonomous and controlled form—in line with the proposition of SDT that an employee can engage in similar behaviors out of different underlying reasons—. We found that an employee who generally perceives organizational pressure to go the extra mile (i.e., citizenship pressure) will be more likely to engage in controlled OCB. Moreover, we found that the consequences of OCB for the employee’s private life depend upon (1) the employee’s motive to engage in OCB and (2) the level of analysis. At the between-person level, autonomously motivated OCB yields positive, whereas controlled motivated OCB yields negative consequences for the employee’s home domain, supporting the SDT propositions. At the within-person level, engaging in autonomous OCB concurrently related to positive as well as to negative consequences for the employee’s home domain, which aligns with the resources investment principle of the Conservation of Resources Theory (Hobfoll, 2001; Lanaj et al., 2016). Overall, our study acknowledges the important role of the employee’s motive to engage in OCB when trying to understand the full consequences of OCB in and outside an employee’s work domain.

## Author Contributions

LG and SDG brainstormed about the research design and collected the data. YG and LG analyzed the data. LG drafted the first manuscript. SDG and YG gave feedback, which was in turn implemented by LG.

## Conflict of Interest Statement

The authors declare that the research was conducted in the absence of any commercial or financial relationships that could be construed as a potential conflict of interest.

## Footnotes

1 Note that the work-home interface is a bi-directional process, with the work domain influencing the home domain and vice versa. However, in this study we focus on the spillover direction from work to home.
