# Supplementary material for: Citizenship Pressure as a Predictor of Daily Enactment of Autonomous and Controlled Organizational Citizenship Behavior: Differential Spillover Effects on the Home Domain
Source: Front Psychol. 2019 Feb 28;10:395. doi: 10.3389/fpsyg.2019.00395 (PMC6404552; doi:10.3389/fpsyg.2019.00395)
Supplement: Supplementary file 1 [file Data_Sheet_1.DOCX]

**Scale development:**

The developmental process of the autonomous and controlled OCB scale started with a review of relevant scientific literature. The initial item pool consisted of items from existing validated OCB measures (i.e., Dalal, Lam, Weiss, Welch, & Hulin, 2009; Podsakoff, Ahearne, & Mackenzie, 1997; Podsakoff & MacKenzie, 1994; Podsakoff, MacKenzie, Moorman, & Fetter, 1990; Smith, Organ, & Near, 1983; Williams & Anderson, 1991). Ambiguously worded, unclearly formulated, and reversed items were excluded because these items were found to correlate positively with measures of counterproductive work behavior (Dalal, 2005). Ten experts in the field of work behavior reviewed the initial item pool to ensure that the items were assessing OCB, could occur and fluctuate on a daily basis, could be performed autonomous as well as controlled, and had face validity. A pilot-study was conducted among a heterogeneous convenience sample of 402 employees working in different sectors. The sample included 61% woman and the average age of the respondents was 38 years (*SD* = 11.67, range: 19-64 years). Around 62.9% of the participants worked as a clerk. These employees filled out the questionnaire consisting of 20 items, once for autonomous OCB and once for controlled OCB. The accompanying scale instruction for autonomous OCB was “Please indicate to what extend you display each of the behaviors below at work 'out of your own initiative', spontaneously, without someone asking you. Answer on a scale ranging from ‘fully disagree’ (1) to ‘fully agree’ (7).” The accompanying scale instruction for controlled OCB was “Please indicate to what extend you display each of the behaviors below at work ‘elicited', because someone expects, urges or explicitly asks you to. Answer on a scale ranging from ‘fully disagree’ (1) to ‘fully agree’ (7).”

To test the dimensionality of our new measure, a confirmatory factor analysis (CFA) was conducted. Overall, our 2-factor model achieved a good fit (χ^2^(689) = 1249.21, *p* < .001, CFI = .93, TLI = .92, SRMR = .06, RMSEA = .05). Moreover, each item loaded significantly and in the expected direction onto its respective latent factor. Additionally, comparing the Bayesian Information Criterion (BIC) values—which represents the balance between the number of parameters (i.e., model complexity) and the fit of the model to the data—of our hypothesized 2-factor model to the alternative single factor model (i.e., all items loading on one OCB factor) revealed that the 2-factor model (BIC = 45599.70) fit the data better than the alternative single factor model (BIC = 499447.35) (Aiken & West, 1991). Note that using Likert scales also allowed a CFA with the classic fit indices and estimators. However, to assess the daily occurrence or absence of autonomous and controlled OCB in our diary study, we altered the 7-point Likert scales to dichotomous response options (for a similar approach see Dalal et al., 2009; Judge et al., 2006). We adhered to this approach because traditional Likert scales typically are used to assess the frequency of OCB enactment over an extended period of time, which is obviously unsuitable for the current study in which participants were surveyed on a daily basis.

Next, the internal consistency of our new scale was examined and we found that autonomous OCB (α = .93) and controlled OCB (α = .97) scale had a satisfactory internal consistency. Moreover, we examined the correlation between the new scale and other established theoretically relevant scales. Autonomous OCB (*r* = .67, *p* < .001) and controlled OCB (*r* = .17, *p* < .05) both correlated positively with the validated OCB scale of Poropat and Jones (2009). Because the conceptualization of autonomous OCB aligns mainly with the original definition and measure of OCB (i.e., voluntary discretionary extra-role behaviors), the convergent validity between autonomous OCB and the validated OCB scale was high as expected, whereas the low correlation between controlled OCB and the validated OCB scale might suggest discriminant validity.

Next, the predictive validity of our new autonomous OCB and controlled OCB scale was examined and we found that they correlated (1) positively with task performance (Williams & Anderson, 1991; *r* = .26, *p* < .001, *r* = 21, *p* < .01, respectively), (2) positively with job satisfaction (Hackman & Oldman, 1991; *r* = .42, *p* < .001, *r* = .25, *p* < .001, respectively), and (3) negatively with turnover intentions (Lum, Kervin, Clark, Reid, & Sirola, 1998; *r* = -.19, *p* < .001, *r* = -.21, *p* < .01, respectively). Note that these correlations were in the expected direction and size as those obtained by previous studies linking traditional scales of OCB to task performance, job satisfaction, and turnover intentions. Moreover, autonomous OCB (*r* = -.13, *ns*), nor controlled OCB (*r* = -.04, *ns*) were significantly related to the social desirability scale of Stöber (2001).

Finally, we found that autonomous OCB and controlled OCB were significantly and positively related with each other (*r* = .37, *p* < .001), indicating that both autonomous OCB and controlled OCB assess the underlying latent variable “OCB”, but that they are not mutually interchangeable constructs (if they were, the correlation would approach unity).

**Scale items:**Indicate whether you displayed each of the behaviors below **today**. Mark the column **'Out of my own initiative**' if you performed the behavior spontaneously, without someone asking you. Mark the column '**Elicited**' if you engaged in the behavior because someone expected, urged or explicitly asked you to. If you did not display the behavior today, mark '**Behavior not performed**'.

|  | **Out of my own initiative** | **Elicited** | **Behavior not performed** |
| --- | --- | --- | --- |
| I took the time to listen to the personal problems of my colleagues/supervisor. | □ | □ | □ |
| In my organization’s interest, I spent time on work-related tasks that are not formally part of my job. | □ | □ | □ |
| I took over tasks from my colleagues/supervisor who were experiencing a high workload. | □ | □ | □ |
| I gave positive feedback to my colleagues/supervisor regarding his/her work. | □ | □ | □ |
| I came to work early and/or stayed late. | □ | □ | □ |
| I performed additional tasks on top of my officially prescribed tasks and/or responsibilities. | □ | □ | □ |
| I behaved in a loyal way towards my organization. | □ | □ | □ |
| I encouraged my colleagues/supervisor. | □ | □ | □ |
| I spoke highly about my organization to others. | □ | □ | □ |
| I oriented new colleagues, although this is not part of my formal duties. | □ | □ | □ |
| I gave work-related advice to my colleagues/supervisor. | □ | □ | □ |
| On top of my own tasks, I shared knowledge and expertise with my colleagues/supervisor. | □ | □ | □ |
| I exerted additional work-related tasks in order to contribute to my organization’s goals. | □ | □ | □ |
| I defended my organization when others criticized it. | □ | □ | □ |
| I helped searching for solutions to the problems my colleagues/supervisor encountered. | □ | □ | □ |
| I assisted my colleagues/supervisor with work-related problems. | □ | □ | □ |
| I suggested constructive ideas to stimulate the functioning of my colleagues/supervisor. | □ | □ | □ |
| I helped my colleagues/supervisor by taken over tasks that do not strictly belong to my own job responsibilities. | □ | □ | □ |
| I took on tasks from my colleagues/supervisor who were absent. | □ | □ | □ |
| I made suggestions and/or gave ideas to stimulate the functioning of my organization. | □ | □ | □ |
